# Supplementary figures and images for: Human non-canonical inflammasomes activate CASP3 to limit intracellular Salmonella replication in macrophages
Source: PLoS Pathog. 2026 Apr 27;22(4):e1014178. doi: 10.1371/journal.ppat.1014178 (PMC13143181; doi:10.1371/journal.ppat.1014178)

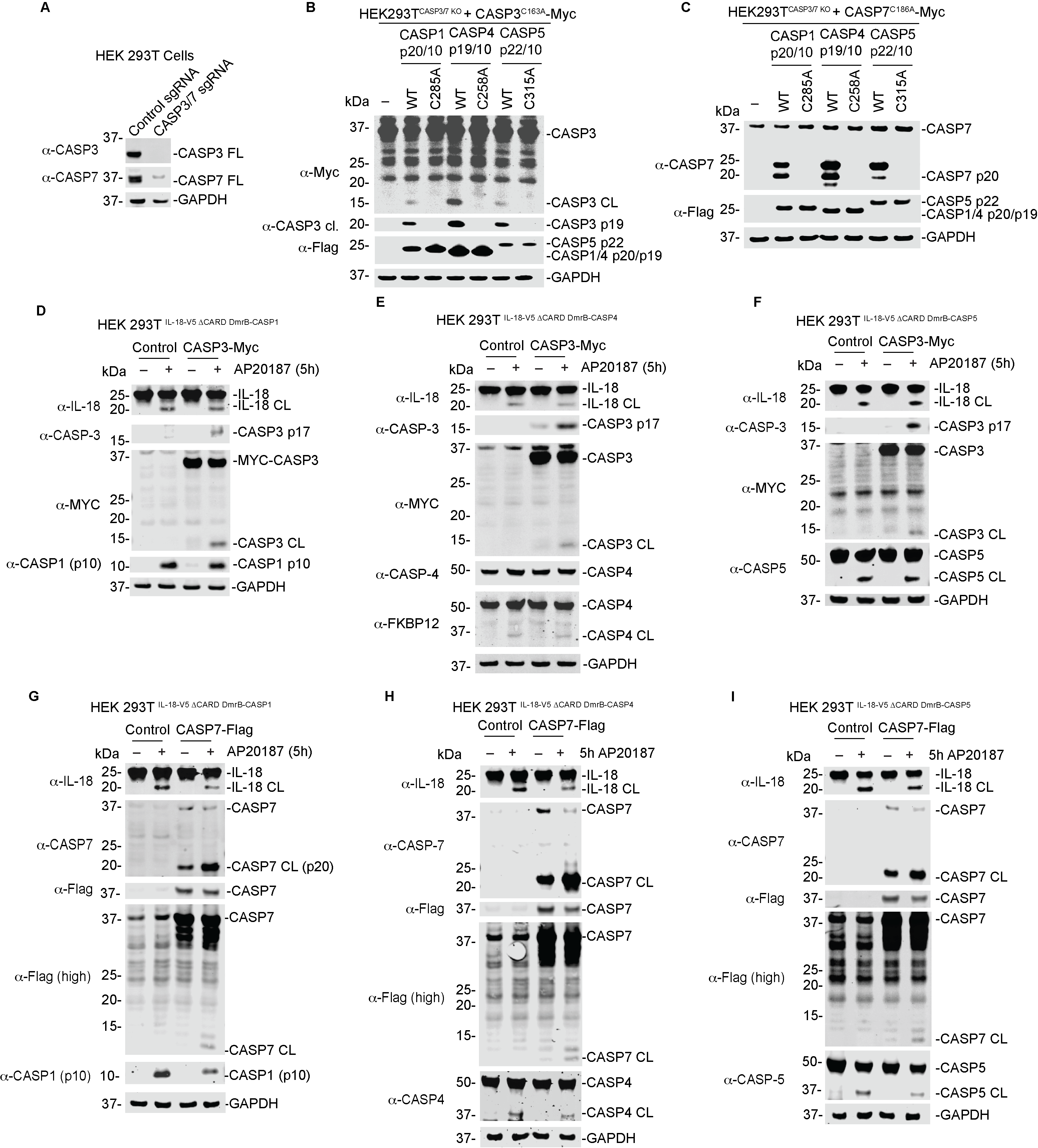

Supplement: S1 Fig — (A) Confirmation of CASP3/7 knockout in HEK 293T cells by immunoblotting. (B,C) CASP3/7 KO HEK 293T cells were transiently transfected with indicated constructs for 24 h then analyzed by immunoblotting. (D-I) HEK 293T cells stably expressing IL-18-V5 and ∆CARD DmrB-CASP1, CASP4, or CASP5 were transiently transfected with indicated constructs coding for C-terminally Myc-tagged CASP3 (CASP3-Myc) (D-F) or C-terminally Flag-tagged CASP7 (CASP7-Flag) (G-I) for 24 h. Cells were then treated with 1 µM AP20187 for 5 h to activate the DmrB-caspases and cell lysates were analyzed by immunoblotting. Data are representative of three or more independent experiments. (TIF) [file ppat.1014178.s001.tif]

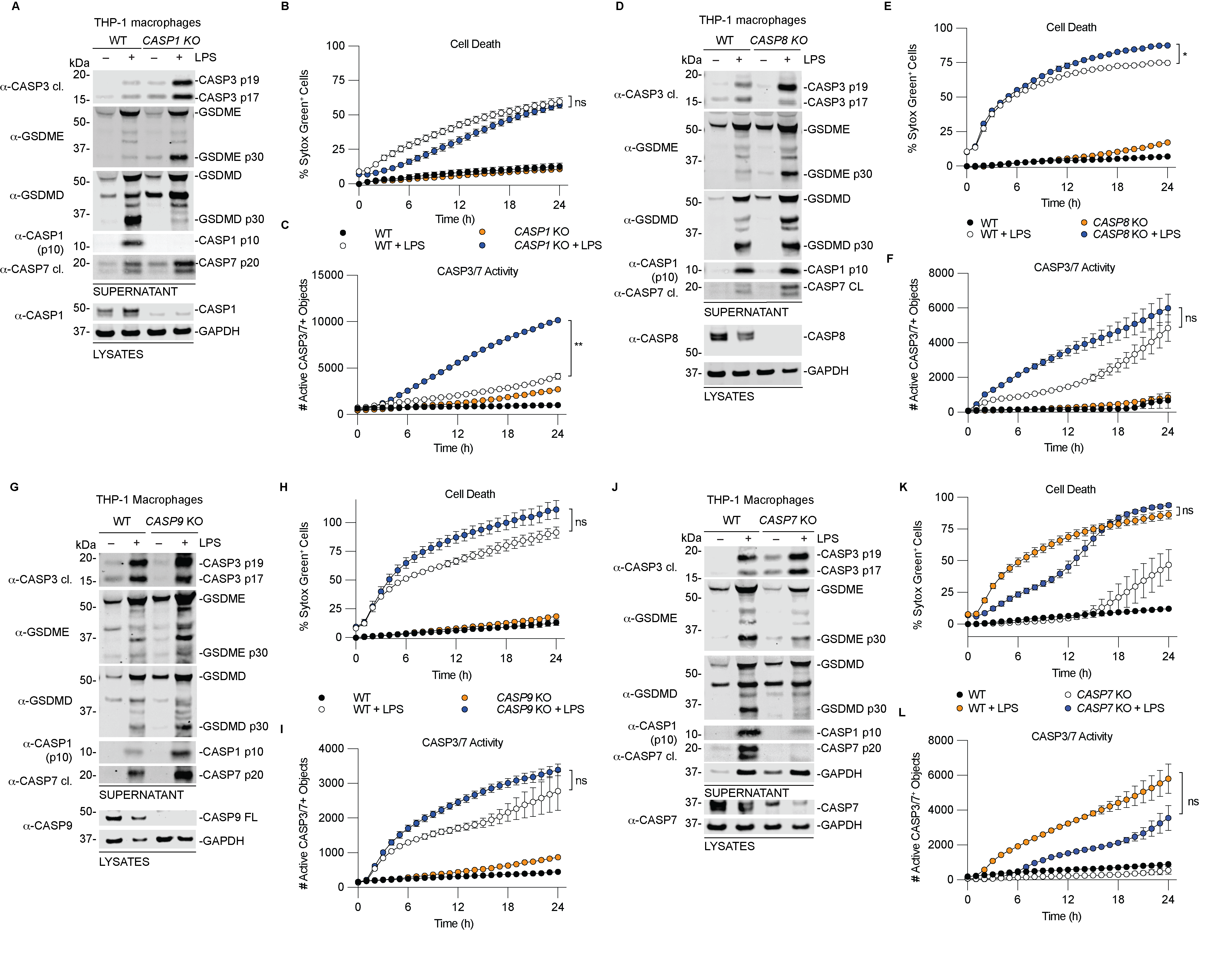

Supplement: S2 Fig — (A-L) Control-matched CASP1 KO (A-C), CASP8 KO (D-F), CASP9 KO (G-I) and CASP7 KO (J-L) THP-1 cells were transfected with 25 µg/mL LPS for 24 h. Supernatants and lysates were then analyzed by immunoblotting (A, D, G, J), cell death was measured by monitoring Sytox Green uptake (B, E, H, K), and CASP3/7 activity was determined using CellTreat CASP3/7 detection reagent (C F, I, L). Data are mean ± SEM of three independent replicates, and representative of at least three independent experiments. ****P < 0.0001, ***P < 0.001, **P < 0.01, and *P < 0.05 by two-way ANOVA test with Tukey’s multiple comparison test comparing the control treated to KO treated samples at 24 h. (TIF) [file ppat.1014178.s002.tif]

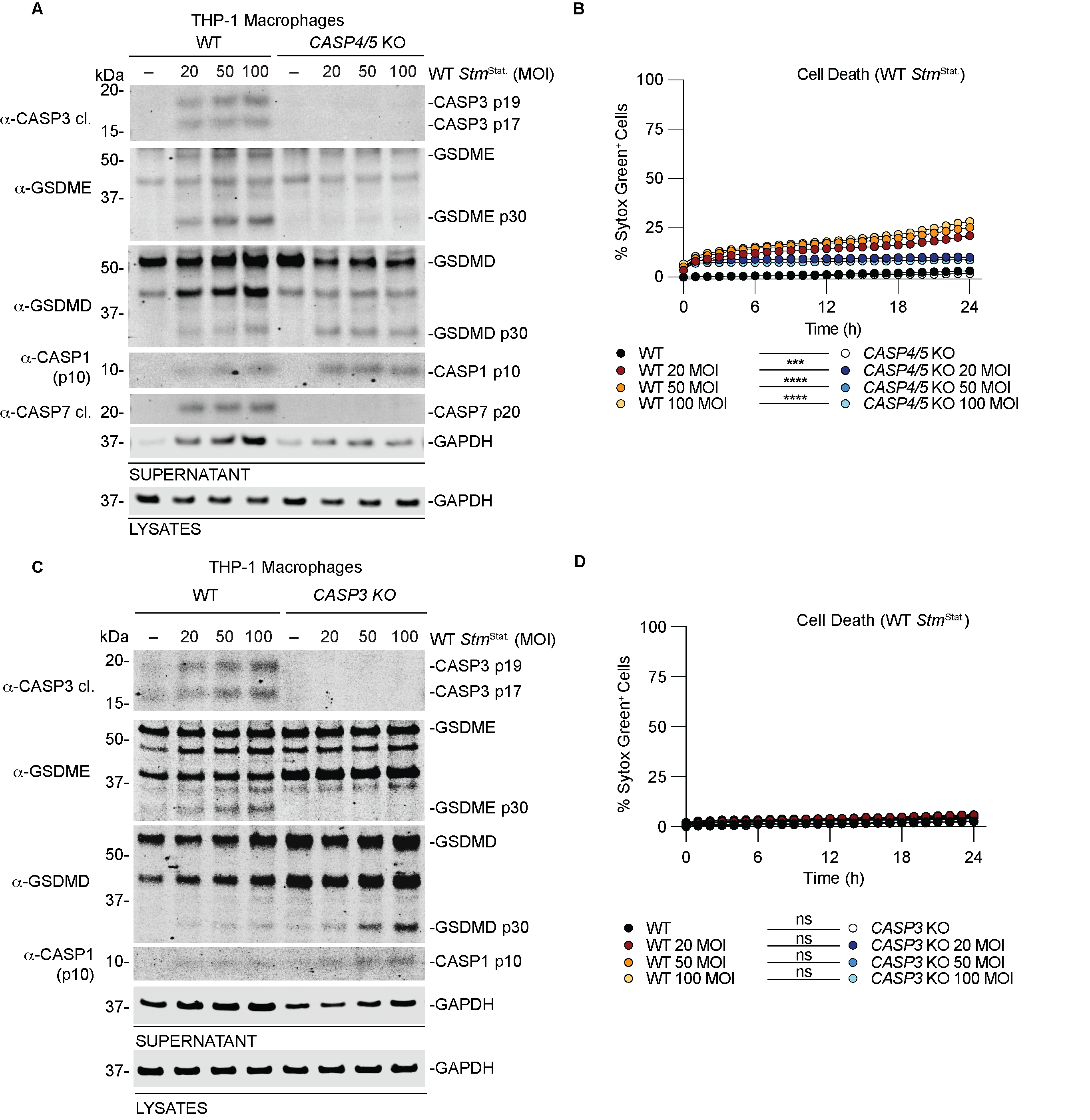

Supplement: S3 Fig — (A-D) WT and CASP4/5 KO (A,B) or WT and CASP3 KO (C,D) THP-1 macrophages were treated with the indicated MOI of WT StmStat. for 24 h. Supernatants and lysates were analyzed by immunoblotting (A,C), and cell death was measured by monitoring Sytox Green uptake (B,D). Data are mean ± SEM of three independent experiments. ****P < 0.0001, ***P < 0.001, **P < 0.01, and *P < 0.05 by two-way ANOVA test with Tukey’s multiple comparison test comparing the control treated to KO treated samples at 24 h. (TIF) [file ppat.1014178.s003.tif]

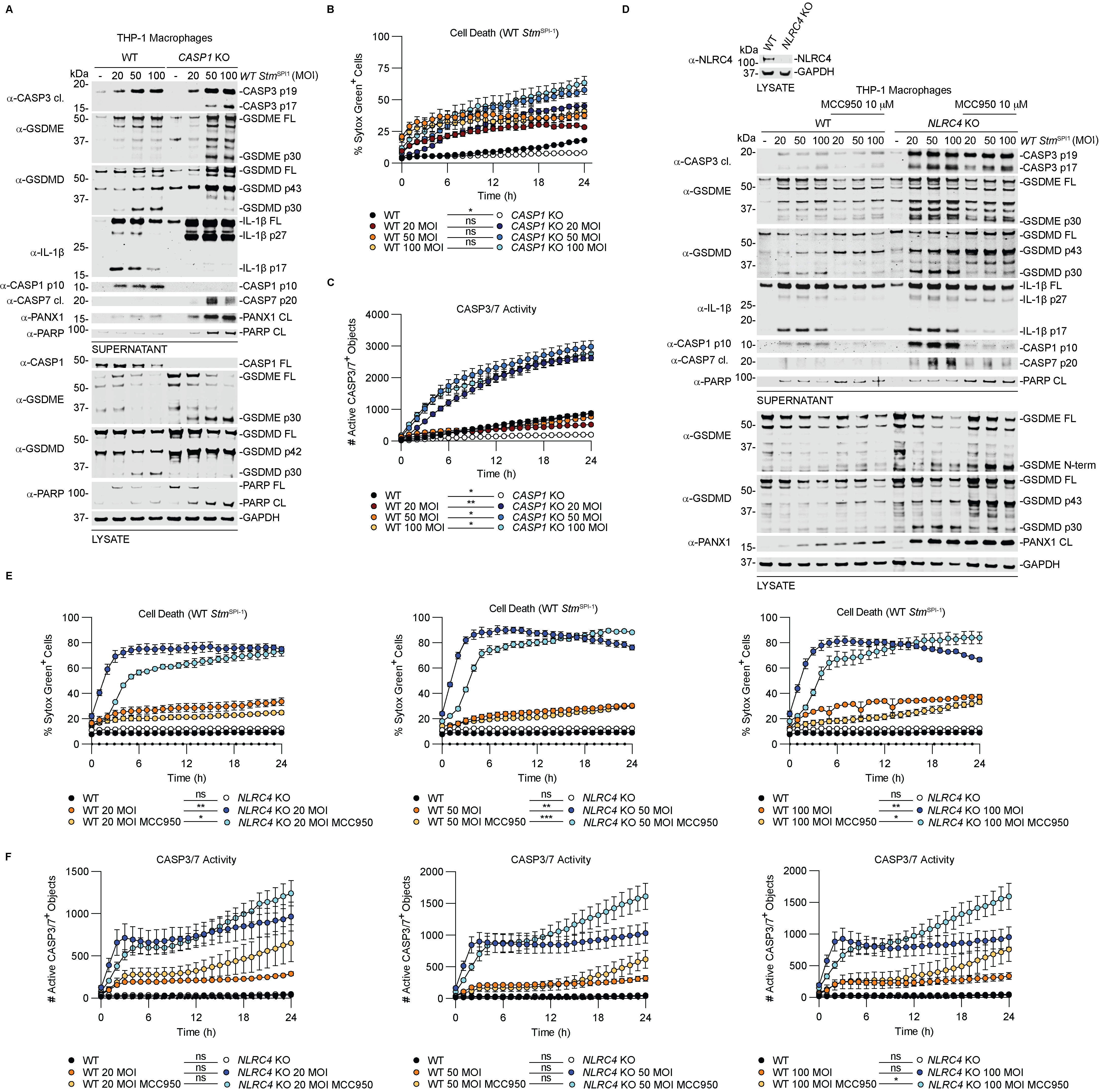

Supplement: S4 Fig — (A-F) WT and CASP1 KO (A-C) or WT and NLRC4 KO THP-1 macrophages (D-F) were treated with the indicated MOI of WT StmSPI-1 for 24 h. Supernatants and lysates were analyzed by immunoblotting (A,D), and cell death was measured by monitoring Sytox Green uptake (B,E), and CASP3/7 activity was determined using CellTreat CASP3/7 detection reagent (C,F). Data are mean ± SEM of three independent replicates, and representative of at least three independent experiments. ****P < 0.0001, ***P < 0.001, **P < 0.01, and *P < 0.05 by two-way ANOVA test with Tukey’s multiple comparison test comparing the control treated to KO treated samples at 24 h. (TIF) [file ppat.1014178.s004.tif]

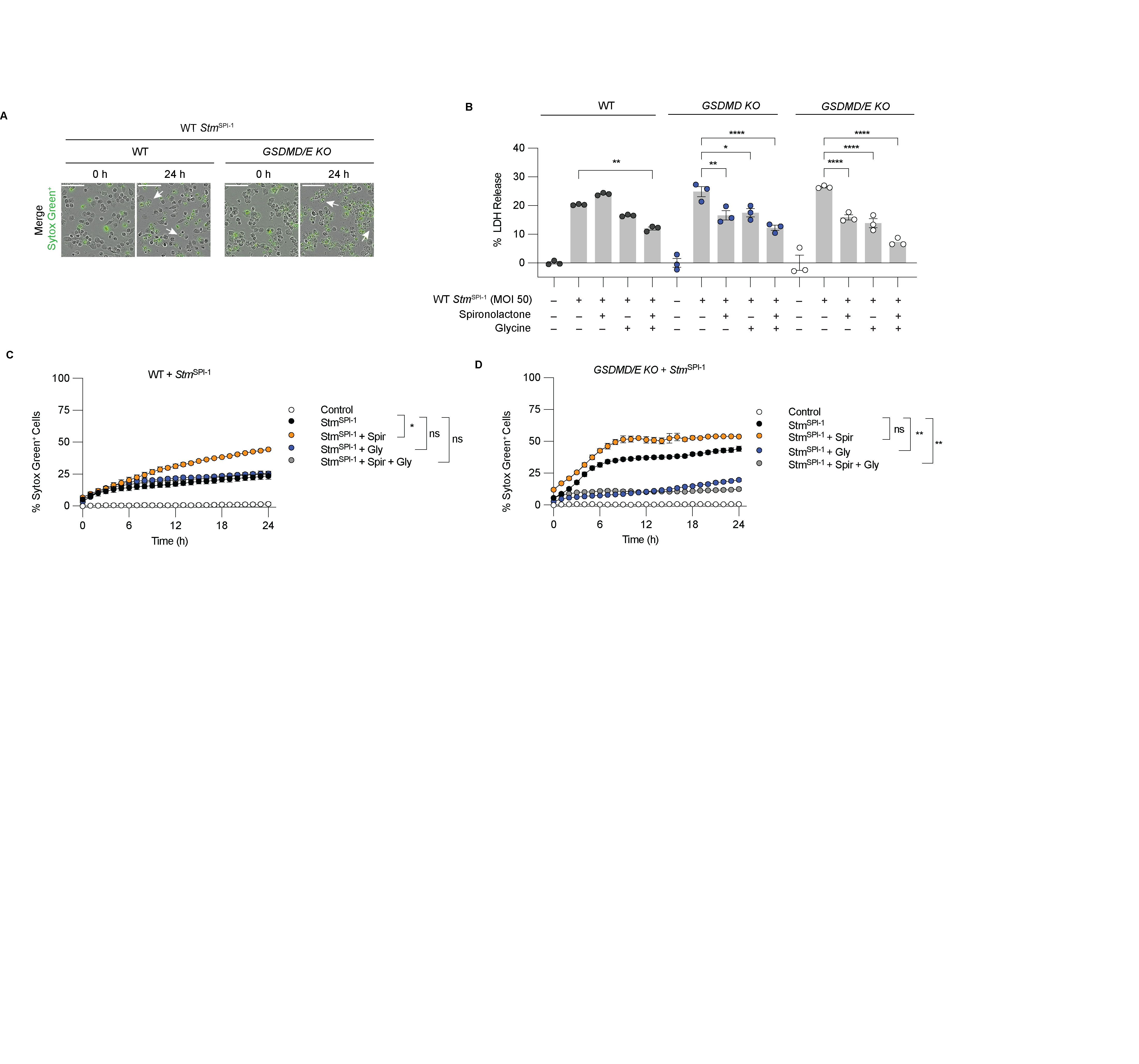

Supplement: S5 Fig — (A) Examples of images of WT and GSDMD/E KO cells at MOI 20 used to quantify Sytox Green uptake are shown. White arrows show pyroptotic morphology and scale bars indicate 10 µm. (B-D) WT, GSDMD KO, and GSDMD/E KO THP-1 macrophages were infected with WT StmSPI-1 at MOI = 50 in the presence or absences of pretreatment with the PANX1 (20 µM spironolactone) or NINJ1 (50 mM glycine) inhibitors for 24 h then LDH release (B) and Sytox Green (C,D) were assessed. Data are mean ± SEM of three independent replicates, and representative of at least three independent experiments.****P < 0.0001, ***P < 0.001, **P < 0.01, and *P < 0.05 by one-way ANOVA test with Tukey’s multiple comparisons test in B or two-way ANOVA test with Tukey’s multiple comparison test comparing the control treated to KO treated samples at 24 h. (TIF) [file ppat.1014178.s005.tif]

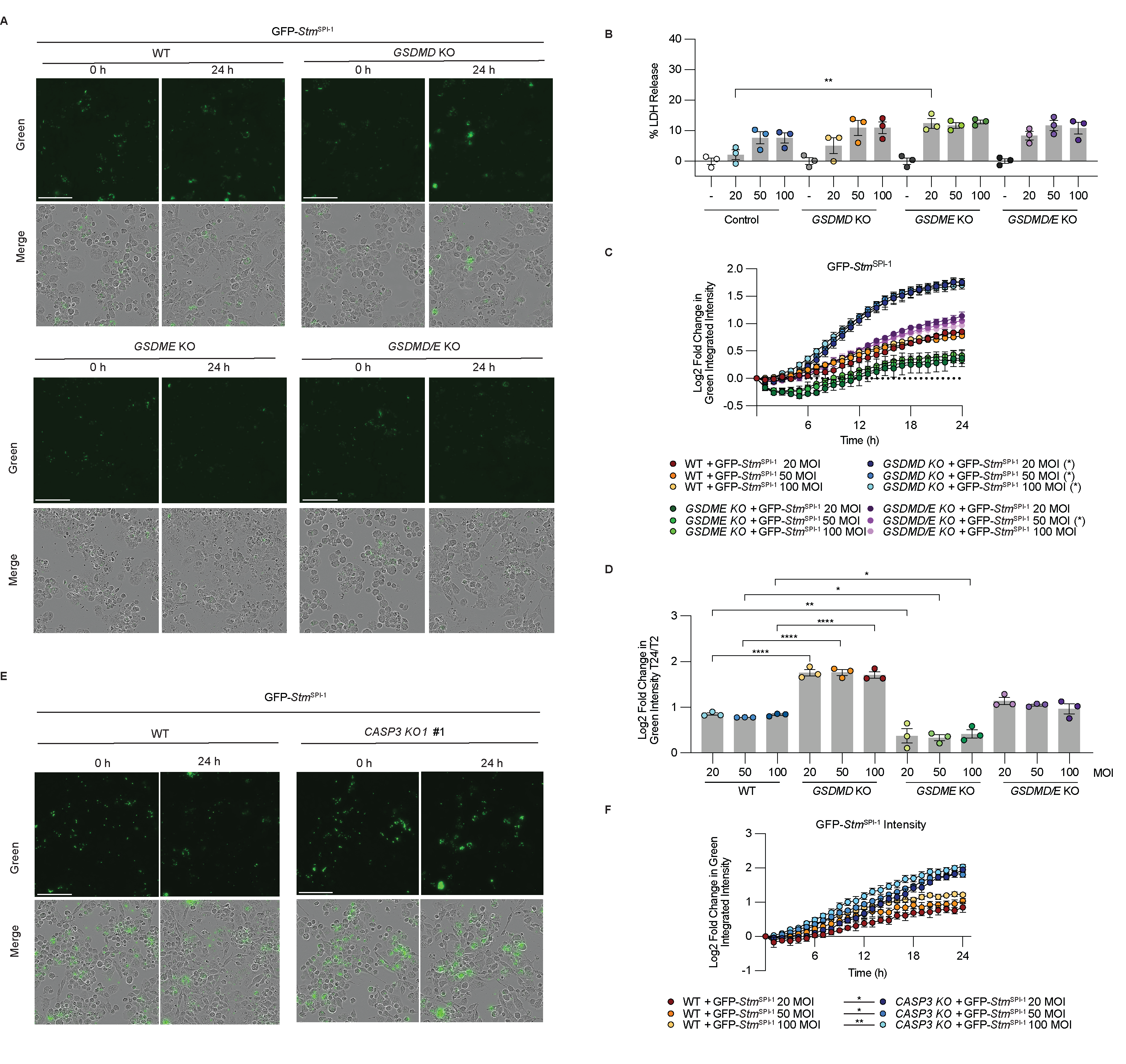

Supplement: S6 Fig — (A-D) WT, GSDMD KO, GSDME KO and GSDMD/E KO THP-1 macrophages were treated with the indicated MOI of GFP-StmSPI-1. Representative images of the GFP-StmSPI-1 (MOI = 20) are shown in A. After 24 h, LDH assays were performed (B). The kinetics of GFP-StmSPI-1replication as measured by the fold change in green intensity is depicted in (C) and the quantification at 24 h is shown in D. Imaging was initiated 2 h post infection (t = 0) using an Incucyte and monitored for 24 h. (E,F) WT or CASP3 KO THP-1 macrophages were treated with the indicated MOI of GFP-StmSPI-1. Representative images of the GFP-StmSPI-1 (MOI = 20) are shown in E. Quantification of the kinetics of the fold change in green intensity is depicted in F. Data are mean ± SEM of three independent replicates, and representative of at least three independent experiments. ****P < 0.0001, ***P < 0.001, **P < 0.01, and *P < 0.05 by one-way ANOVA test with Tukey’s multiple comparison’s test in B, D or two-way ANOVA test with Tukey’s multiple comparison test comparing the control treated to KO treated samples at 24 h. (TIF) [file ppat.1014178.s006.tif]
