# Supplementary material for: Human non-canonical inflammasomes activate CASP3 to limit intracellular Salmonella replication in macrophages
Source: PLoS Pathog. 2026 Apr 27;22(4):e1014178. doi: 10.1371/journal.ppat.1014178 (PMC13143181; doi:10.1371/journal.ppat.1014178)
Supplement: Attachment — Submitted filename: Response to Reviewers.docx [file ppat.1014178.s008.docx]

PPATHOGENS-D-25-02867
Human non-canonical inflammasomes activate CASP3 to limit intracellular Salmonella replication in macrophages
PLOS Pathogens

Dear Dr. Taabazuing,

Thank you for submitting your manuscript to PLOS Pathogens. After careful consideration, we feel that it has merit but does not fully meet PLOS Pathogens's publication criteria as it currently stands. Therefore, we invite you to submit a revised version of the manuscript that addresses the points raised during the review process.

Please submit your revised manuscript by Mar 06 2026 11:59PM. If you will need more time than this to complete your revisions, please reply to this message or contact the journal office at [plospathogens@plos.org](mailto:plospathogens@plos.org). When you're ready to submit your revision, log on to <https://www.editorialmanager.com/ppathogens/> and select the 'Submissions Needing Revision' folder to locate your manuscript file.

Please include the following items when submitting your revised manuscript:

* A letter that responds to each point raised by the editor and reviewer(s). You should upload this letter as a separate file labeled 'Response to Reviewers'. This file does not need to include responses to any formatting updates and technical items listed in the 'Journal Requirements' section below.
* A marked-up copy of your manuscript that highlights changes made to the original version. You should upload this as a separate file labeled 'Revised Manuscript with Track Changes'.
* An unmarked version of your revised paper without tracked changes. You should upload this as a separate file labeled 'Manuscript'.

If you would like to make changes to your financial disclosure, competing interests statement, or data availability statement, please make these updates within the submission form at the time of resubmission. Guidelines for resubmitting your figure files are available below the reviewer comments at the end of this letter.

We look forward to receiving your revised manuscript.

Kind regards,

Dana J. Philpott
Academic Editor
PLOS Pathogens

Eva Heinz
Section Editor
PLOS Pathogens

Sumita Bhaduri-McIntosh
Editor-in-Chief
PLOS Pathogens
orcid.org/0000-0003-2946-9497

Michael Malim
Editor-in-Chief
PLOS Pathogens
orcid.org/0000-0002-7699-2064

**Additional Editor Comments:**

Thank you for submitting your work to PLOS Pathogens. The Reviewers were quite positive about the work but have some concerns that will need to be addressed. In particular, there were comments related to the timing of cleavage of substrates in the Western blots and recommendations to validate some of the work with recombinant proteins to prove specificity. Please provide a detailed point-by-point response to each comment.


**Journal Requirements:**

1) Please ensure that the CRediT author contributions listed for every co-author are completed accurately and in full.
At this stage, the following Authors/Authors require contributions: Madhura Kulkarni, Christopher Bourne, Ashutosh Mahale, Patrick Exconde, Cecelia Murphy, Sofia Cervantes, Matilda Kardhashi, Mirai Kambayashi, William Yoo, Tristan Wrong, Robert Patio, Bohdana Discher, and Cornelius Taabazuing. Please ensure that the full contributions of each author are acknowledged in the "Add/Edit/Remove Authors" section of our submission form.
The list of CRediT author contributions may be found here: <https://journals.plos.org/plospathogens/s/authorship#loc-author-contributions>

2) We ask that a manuscript source file is provided at Revision. Please upload your manuscript file as a .doc, .docx, .rtf or .tex. If you are providing a .tex file, please upload it under the item type u2018LaTeX Source Fileu2019 and leave your .pdf version as the item type u2018Manuscriptu2019.

3) We do not publish any copyright or trademark symbols that usually accompany proprietary names, eg ©,  ®, or TM  (e.g. next to drug or reagent names). Therefore please remove all instances of trademark/copyright symbols throughout the text, including:
- ® on page: 16 and 20.
- TM on page: 16 and 21..

4) Please upload all main figures as separate Figure files in .tif or .eps format. For more information about how to convert and format your figure files please see our guidelines: 
<https://journals.plos.org/plospathogens/s/figures>

5) We have noticed that you have uploaded Supporting Information files, but you have not included a list of legends. Please add a full list of legends for your Supporting Information files after the references list.

6) In the online submission form, you indicated that "Further information and requests for resources and reagents should be directed to and will be fulfilled by the lead contact, Cornelius Y. Taabazuing ([Cornelius.taabazuing@pennmedicine.upenn.edu](mailto:Cornelius.taabazuing@pennmedicine.upenn.edu))". All PLOS journals now require all data underlying the findings described in their manuscript to be freely available to other researchers, either
1. In a public repository
2. Within the manuscript itself
3. Uploaded as supplementary information.
This policy applies to all data except where public deposition would breach compliance with the protocol approved by your research ethics board. If your data cannot be made publicly available for ethical or legal reasons (e.g., public availability would compromise patient privacy), please explain your reasons by return email and your exemption request will be escalated to the editor for approval. Your exemption request will be handled independently and will not hold up the peer review process, but will need to be resolved should your manuscript be accepted for publication. One of the Editorial team will then be in touch if there are any issues.

All the data for this manuscript are available within the manuscript. This statement was to inform readers that if they need resources such as cell lines generated during this work, we will freely share them upon request.

7) Please amend your detailed Financial Disclosure statement. This is published with the article. It must therefore be completed in full sentences and contain the exact wording you wish to be published.
- State the initials, alongside each funding source, of each author to receive each grant. For example: "This work was supported by the National Institutes of Health (####### to AM; ###### to CJ) and the National Science Foundation (###### to AM)."
- State what role the funders took in the study. If the funders had no role in your study, please state: "The funders had no role in study design, data collection and analysis, decision to publish, or preparation of the manuscript.".
If you did not receive any funding for this study, please simply state: u201cThe authors received no specific funding for this work.u201d

**Reviewers' Comments:**

Reviewer's Responses to Questions

**Part I - Summary**
Please use this section to discuss strengths/weaknesses of study, novelty/significance, general execution and scholarship.

Reviewer #1: This paper is a detailed analysis of the role of non-canonical inflammasomes in activating CASP3. The important result of the paper is that in human cells, CASP4/5 can cleave CASP3. CASP3 seems to play a small role in restriction of Salmonella replication but how it does so remains unclear. Overall I feel the paper will be of some interest to specialists interested in the details of pathogen-induced cell death.

We thank the reviewer for their positive assessment. We agree that the mechanism by which CASP3 restricts Salmonella replication remains an important open question. In the revised manuscript, we have expanded the Discussion to address this point. Specifically, we now discuss the possibility that CASP3 restricts bacterial replication through cleavage of host or bacterial effector proteins, rather than through cell death alone. This is supported by our observation that cell death is unchanged in CASP3 KO cells despite increased bacterial replication.

Reviewer #2: In this study, the authors explore the links between inflammatory caspases and their substrates in the control of Salmonella replication. The data presented combines biochemistry and genetics in an impressive manner, leading to simple interpretation of the data presented. As Salmonella induced cell death, and its impact on intracellular replication, have been heated topics of investigation for many years, this study will likely attract significant attention from the community. With this expectation in mind, the following suggestions are offered to improve the quality of the data supporting the conclusions offered. Point 2 is the most important to address.

1. Line 80 describes the authors report that caspase-4/5 cleaves pro-IL-1 and pro-IL-18. The author should cite the prior studies by Shao and Kagan on this topic.

We thank the reviewer for pointing this out, we have included the citations.

2. Although the caspase substrate cleavage assays presented throughout this study are impressively clean (with appropriate controls), the length of time used in these assays atypical. The 1-3 hours of cleavage time is not appropriate for the authors to establish the efficiency of cleavage of each substrate of interest. The authors are encouraged to perform assays using recombinant proteins, as has been established by the labs indicated in point 1 above, in order to determine the relative efficiency by which caspase-7 is cleaved. This suggestion, while important for the authors are consider, should not be taken as a requirement to replace all assays in this study with those using recombinant proteins. However, some key experiments would be important to include in a revised manuscript.

We thank the reviewer for this suggestion and we agree that demonstrating direct cleavage is important. In the revised manuscript, we have strengthened this point. We introduced cleavage-resistant mutants (CASP3 D175A and CASP7 D198A) and show that in purified in vitro assays, these mutations abolish processing by inflammatory caspases (Fig. 1B,C). This demonstrates that cleavage occurs at canonical activation sites.

This manuscript was done in collaboration with the Boucher lab and they have performed recombinant assays using purified proteins and determined the efficiency of CASP3/7 relative to other substrates (Sebastian Grant et al. Biorxiv 2025 https://doi.org/10.1101/2025.11.01.685735). They observed that CASP3/7 are slow relative to other well established substrates using these in vitro assays. However, in the cell, where multiple substrates are present at different subcellular localizations and abundance, this can shift the preference as our data suggests. We observe that when all substrates are present in lysates (Fig. 2D) CASP4 is better at cleaving IL-18 compared to CASP1. This may also differ based on the cell type. For example, CASP4 can efficiently cleave GSDMD in epithelial cells and is the major driver of LPS induced death in that context whereas CASP1 appears to be the dominant protease in macrophages. Thus, substrate abundance, localization, and competition in cells likely influence cleavage efficiency in cells.

3. Figure 3A is confusing, as the authors show that caspase-4/5 double knockout cells retain the ability to cleave GSDMD after LPS treatment. As current literature suggests that caspase-4/5 would be the only pathway to GSDMD cleavage, the authors are encouraged to explore this observation further.

We thank the reviewer for pointing this out. We were also surprised by this observation in 2023 when we initially published the *CASP4/5* KO cells and made this observation. This suggested to us that in human cells, there exists a receptor for LPS other than CASP4/5. Based on the literature, we hypothesized that this receptor is NLRP11 and had initially mentioned it in our discussion but were asked to remove it by the reviewers of that manuscript. NLRP11 was reported to work with NLRP3 to activate CASP1 (Gangopadhyay et al. Nature Immunology 2022, Rojas-Lopez et al. Science Immunology 2023). We think NLRP11 is activating NLRP3 to activate CASP1 in the absence of CASP4/5 and this is the subject of future studies in the lab. However, it’s beyond the scope of this paper. We have added this to the discussion.

4. The acronym Salmonella enterica serovar Typhimurium (Stm) is described multiple times in this manuscript. Please adjust for one description.

We thank the reviewer for noting this and have made the necessary adjustment.

Reviewer #3: The study by Kulkarni et al. used human macrophages to characterize the role played by CASP4/5 in the cleavage of CASP3, which in turn leads to the cleavage of GSDME, and this pathway was functional in response to both intracellular LPS as well as infection with Salmonella.
Overall, this is an interesting and carefully performed study that clarifies the contributions of GSDMD-dependent pyroptosis versus CASP3-dependent functions (either apoptosis or GSDME-dependent pore formation) in human macrophages.

**Part II – Major Issues: Key Experiments Required for Acceptance**
Please use this section to detail the key new experiments or modifications of existing experiments that should be absolutely required to validate study conclusions.

Generally, there should be no more than 3 such required experiments or major modifications for a "Major Revision" recommendation. If more than 3 experiments are necessary to validate the study conclusions, then you are encouraged to recommend "Reject".

Reviewer #1: In terms of concrete suggestions to improve the manuscript, I think there are two major issues that should be addressed:

1. The claim that CASP3/7 are “direct” substrates of CASP4/5 needs experiments in which both the enzyme (CASP4 or 5) and the substrate (CASP3 or 7) are purified recombinant proteins. Unless I missed something, the experiments performed used cell lysates expressing CASP3/7 instead of purified CASP3/7. I believe that the presence of other proteins in the lysate creates the possibility that cleavage of CASP3/7 is indirect. In addition, some enzymology (kcat/Km) to show that the cleavage is efficient (comparable to other bona fide substrates) would bolster the case that these are indeed truly direct substrates and not simply being cleaved during non-physiological experimental conditions. I also believe that the cleavage site should be identified, either by MS or by mutating the site and showing cleavage no longer occurs.

We thank the reviewer for this suggestion and we agree that demonstrating direct cleavage is important. In the revised manuscript, we have strengthened this point. We introduced cleavage-resistant mutants (CASP3 D175A and CASP7 D198A) and show that using purified enzymes, these mutations abolish processing by inflammatory caspases (Fig. 1B,C). This demonstrates that cleavage is direct and occurs at canonical activation sites.

This manuscript was done in collaboration with the Boucher lab and they have performed recombinant assays using purified proteins and determined the efficiency of CASP3/7 relative to other substrates (Sebastian Grant et al. Biorxiv 2025 https://doi.org/10.1101/2025.11.01.685735). They observed that CASP3/7 are slow relative to other well established substrates. However, we believe that substrate abundance, localization, and competition in cells likely influence cleavage efficiency in cells. For example, we observe that when all substrates are present in lysates (Fig. 2D) and the enzymes are present at equivalent activity units, CASP4 is better at cleaving IL-18 compared to CASP1. This may also differ based on the cell type. For example, CASP4 can efficiently cleave GSDMD in epithelial cells and is the major driver of LPS induced death in that context whereas CASP1 appears to be the dominant protease in macrophages.

2. The claim that CASP1/8/9 are dispensable for CASP3/7 activation needs to be confirmed in CASP1/8/9 triple KO cells. Although individual KO are tested, the possibility remains that there is compensation/redundancy.

We agree with the reviewer and have addressed this directly. We generated CASP1/CASP8/CASP9 triple knockout THP-1 cells and tested CASP3/7 activation following LPS transfection. We observed robust CASP3/7 cleavage and activity in triple knockout cells, with no reduction in cell death. These data demonstrate that CASP1, CASP8, and CASP9 are dispensable for CASP3/7 activation during non-canonical inflammasome signaling. These results are now included in Fig. 2.

Reviewer #2: (No Response)

Reviewer #3: In Fig. 3A, there is still a relatively substantial cleavage of GSDMD observed, as well as CASP1 p10 formation, in CASP4/5 DKO cells in response to intracellular LPS. Given that CASP4/5 are the sensors of intracellular LPS, how can this be explained? Is this processing driven by TLR4? Using TLR4 KO cells would clarify this point.

This is a great point made by the reviewer. We do not think that this is driven by TLR4, we are not priming (although residual LPS could) in our assays. NLRP11 was reported to work with NLRP3 to activate CASP1 (Gangopadhyay et al. Nature Immunology 2022, Rojas-Lopez et al. Science Immunology 2023). We think NLRP11 is acting as an LPS sensor in the absence of CASP4/5 and activating NLRP3 to activate CASP1. We have added this discussion to the revised manuscript.

The authors used Salmonella grown to stationary phase to “to minimize the contribution of the canonical caspase-1 signaling pathway” (line 258). This is not very clear and the authors should explain what the growth conditions is expected to do. Is it only to limit expression of the TTSS and thereby NLRC4 detection? Or also to limit NLRP3? Or both? But isn’t the Salmonella strain used flagellated? This would likely also drive NLRC4. Using flagellated versus non-flagellated strains could solve this point.

We thank the reviewer for pointing out that clarification is needed on this point. Salmonella has been reported to activate both NLRP3 and NLRC4 (Naseer et al. Plos Pathogens 2022). We are using flagellated Salmonella which would be sensed by both inflammasomes. Using non-flagellated Salmonella would likely come with a great fitness disadvantage, resulting in decreased infections. In the revised manuscript we now use CASP1 KO, NLRC4 KO, and NLRC4 KO cells treated with MCC950 to inhibit NLRP3, effectively removing any contributions from the canonical pathway. In this context, we still observe CASP3/7 processing, suggesting the non-canonical inflammasomes are mediating their activation. These results are now included as supplemental figure 4.

In Fig 5A-B, the results obtained with GSDME KO macrophages are puzzling. I wonder if different results would have been obtained if the initial time against which everything is measured was earlier than 2h… Indeed, 2h is already a very long time when considering pyroptotic events, which occur in minutes.

We thank the reviewer for pointing this out and agree that 2 hours is already a late time point to start measuring but this is a technical limitation with how the infections are conducted. We infect for 1 hour, then treat with gentamycin to kill the extracellular bacteria for another hour, then start measuring. We do note that there is already some death as indicated by time zero starting at 20% Sytox Green in some instances. In the revised manuscript we now include the kinetics of Salmonella replication over time in gasdermin and CASP3 deficient cells in supplemental figure 6. We see that for GSDMD KO cells, the increase in replication starts around 6 hours and for CASP3 KO cells, this starts around 12 hours. It’s unlikely that we would have observed differences in GSDME KO cells being able to control replication had we looked earlier.

**Part III – Minor Issues: Editorial and Data Presentation Modifications**
Please use this section for editorial suggestions as well as relatively minor modifications of existing data that would enhance clarity.

Reviewer #1: Minor points:

line 18 (abstract) -- reword so as not to imply that cleavage of IL-1/18 induces pyroptosis

We thank the reviewer for pointing this out and have now revised it to imply cleavage of GSDMD induces pyroptosis.

line 24-26 (abstract) -- I find this sentence to be confusing. It is not obvious to me how it follows from the fact that CASP1 is responsible for GSDMD cleavage that GSDMD not GSDME is the principal driver of pyroptosis

We thank the reviewer for pointing this out. We find that during LPS transfection, GSDMD KO, but not GSDME KO cells, had a defect in LDH release but cell lysis can occur independently of gasdermin proteins in the context of bacterial infection. We have now clarified this in the abstract.

Line 140 - Fig. 1F (not Fig. 1C)

We thank the reviewer for their careful reading and have made the correction.

Figure 3B/E/H/K -- why does cell death of control cells reach 100% in some experiments, but only 25-30% in others?

In our experiments, we use cell lines obtained from different labs and those that were generated using different methods. Therefore, we use the matched controls from those labs or cells generated and the control cells behaved slightly differently. Some were very responsive and some not as responsive.

Figure 4 -- why do GSDME KO cells die more in Salmonella infection than controls? This deserves some explanation, especially given that in Fig 3, the GSDME KO cells are defective for cell death in response to LPS.

In figure 3, GSDMD KO, but not GSDME KO cells were defective for LPS death, and GSDMD/E KO phenocopied GSDMD KO cells. We observe a difference in the requirement for gasdermins during LPS and Salmonella infections. With LPS GSDMD appears to be the dominant player but in the context of Salmonella, gasdermin independent pathways seem to contribute to cell death. Our data suggest that NINJ1 and PANX1 likely mediate this death. The kinetics of this gasdermin-independent cell lysis have not been evaluated relative to the gasdermin-dependent pathways. We observe an increase in GSDMD processing in GSDME KO cells, along with robust activation of CASP3/7 relative to WT cells, which could drive the PANX1/NINJ1 mediated lysis. It appears that in the absence of one of the gasdermins, the other pathway to cleave the other gasdermin becomes favored. As GSDMD is a key mediator of cell lysis, this explains why we might see more death in GSDME KO cells.

Reviewer #2: (No Response)

Reviewer #3: Lines 105-6: I don’t understand why the lack of human CASP3 cleavage by CASP11 “aligns” with data on the cleavage of mIL18. It just looks like two totally unrelated facts.

We agree with the reviewer and have removed this.

Line 161: Is the effect of MCC950 on CASP3/GSDME driven by the lack of activation of CASP1 or the lack of activation of GSDMD?

We thank the reviewer for noting this point of clarification. We meant that it could facilitate pathogen dissemination through unknown mechanisms. We removed the speculative statement regarding pathogen entry.

Lines 241-48: what about the release of active IL18?

We agree with the reviewer that this would add value but detection sensitivity limited our ability to measure IL-18. The values were around the limit of detection for the assay and we could not confidently stand by the results.

Line 460: “if GSDME pores are sublytic, as has been suggested, it could facilitate pathogen entry” I think the authors should clarify what they mean here. One may think that this would suggest that the bacteria could enter through the GSDME pores, which is certainly not realistic.

We thank the reviewer for noting this point of clarification. We meant that it could facilitate pathogen dissemination through unknown mechanisms. We removed the speculative statement regarding pathogen entry.

PLOS authors have the option to publish the peer review history of their article ([what does this mean?](https://track.editorialmanager.com/CL0/https:%2F%2Fjournals.plos.org%2Fplospathogens%2Fs%2Feditorial-and-peer-review-process%23loc-peer-review-history/1/010f019b8f80ed00-b47b2de6-394d-4d6c-bbcf-5f023e1d5056-000000/ajZ4xWIwyETPi3E2Elm10DLHp8HoHdna1nQIgXV7qUQ=244)). If published, this will include your full peer review and any attached files.


If you choose “no”, your identity will remain anonymous but your review may still be made public.


**Do you want your identity to be public for this peer review?** For information about this choice, including consent withdrawal, please see our [Privacy Policy](https://track.editorialmanager.com/CL0/https:%2F%2Fwww.plos.org%2Fprivacy-policy/1/010f019b8f80ed00-b47b2de6-394d-4d6c-bbcf-5f023e1d5056-000000/fyfxZ7qB-ykh0derziGNyhwnr9wL_nHuqJK3Io9xzwM=244).

Reviewer #1: No

Reviewer #2: No

Reviewer #3: No

[NOTE: If reviewer comments were submitted as an attachment file, they will be attached to this email and accessible via the submission site. Please log into your account, locate the manuscript record, and check for the action link "View Attachments". If this link does not appear, there are no attachment files.]

**Figure resubmission:**

While revising your submission, we strongly recommend that you use PLOS’s NAAS tool (<https://ngplosjournals.pagemajik.ai/artanalysis>) to test your figure files. NAAS can convert your figure files to the TIFF file type and meet basic requirements (such as print size, resolution), or provide you with a report on issues that do not meet our requirements and that NAAS cannot fix.

After uploading your figures to PLOS’s NAAS tool - <https://ngplosjournals.pagemajik.ai/artanalysis>, NAAS will process the files provided and display the results in the "Uploaded Files" section of the page as the processing is complete. If the uploaded figures meet our requirements (or NAAS is able to fix the files to meet our requirements), the figure will be marked as "fixed" above. If NAAS is unable to fix the files, a red "failed" label will appear above. When NAAS has confirmed that the figure files meet our requirements, please download the file via the download option, and include these NAAS processed figure files when submitting your revised manuscript.

**Reproducibility:**

To enhance the reproducibility of your results, we recommend that authors of applicable studies deposit laboratory protocols in protocols.io, where a protocol can be assigned its own identifier (DOI) such that it can be cited independently in the future. Additionally, PLOS ONE offers an option to publish peer-reviewed clinical study protocols. Read more information on sharing protocols at <https://plos.org/protocols?utm_medium=editorial-email&utm_source=authorletters&utm_campaign=protocols>
